# Hypertension and Diabetes Mellitus among Patients at Hawassa University Comprehensive Specialized Hospital, Hawassa, Southern Ethiopia

**DOI:** 10.1155/2019/2509242

**Published:** 2019-04-08

**Authors:** Andargachew Kassa, Endrias Markos Woldesemayat

**Affiliations:** ^1^College of Medicine and Health Sciences, School of Nursing and Midwifery, Hawassa University, Ethiopia; ^2^College of Medicine and Health Sciences, School of Public and Environmental Health, Hawassa University, Ethiopia

## Abstract

**Background:**

The burden of noncommunicable disease (NCD) in Africa is on a remarkable rise exacerbating the poor public health status affected by the existing but yet unsolved communicable disease. In Ethiopia, there is a paucity of evidence regarding prevalence and risk factors to NCD.

**Objective:**

This study sought to determine the prevalence of risk factors of NCDs, prevalence of DM and HTN, and risk factors associated with diabetes mellitus (DM) and hypertension (HTN).

**Method:**

This is an institution based cross-sectional study conducted on a sample of 411 clients attending a university-based comprehensive specialized hospital in Southern Ethiopia. The data was collected by using a pretested interviewer-administered questionnaire and observational checklist. Frequency, proportions, bivariate and multivariate logistic regression analysis was conducted using SPSS software version 20.

**Result:**

We identified 64.2% of the clients had at least one of the risk factors to the NCDs. One-third (33.3%) had physical inactivity, whereas 20.2% had a BMI of ≥ 25%. The prevalence of DM and HTN was 12.2% and 10.5%, respectively. The multivariate analysis demonstrated that age ≥ 60 years, physical inactivity, higher BMI, and cigarette smoking were risk factors for at least one of the NCDs.

**Conclusion:**

The prevalence of DM and prevalence of HTN were high. The magnitudes of risk factors to NCDs among the study population were substantial. Higher BMI, physical inactivity, low fruit and vegetable consumption, alcohol use, khat chewing, and cigarette smoking were among the prevailing risk factors identified.

## 1. Introduction

In the developing world, communicable diseases were the major causes of morbidity and mortality and still, they continued to be the cause. But noncommunicable diseases (NCDs) are on a remarkable rise causing ill health and death for millions. NCD is the cause of 63% of annual global death report, of which 14 million deaths are premature. Unfortunately, 86% of the premature deaths are happening in the middle- and lower-income countries (LMICs) [1]. NCDs are happening as a result of demographic transition characterized by urbanization, industrialization, and the ever-improving life expectancy noted in these countries [2]. The expansion of these catastrophic NCD started exacerbating the existing poor health status of the public and also enticing the delivery of health care service [2, 3]. The WHO has identified four major NCDs happening in the developing region. These are cardiovascular diseases (CVD), cancer, chronic respiratory diseases, and diabetes (DM) [2].

The common risk factors for these four NCDs are classified into two categories called behavioral and biomedical risk factors. By avoiding the behavioral risk factors such as tobacco use, physical inactivity, unhealthy dietary practice, and alcohol abuse, it is possible to reduce 80% of CVDs and DM. In addition, one-third of all forms of cancers can be prevented by avoiding these risk factors. Unless these behavioral risk factors are avoided, the development of other formidable biomedical risk factors such as increments in blood pressure, blood sugar, blood lipids, and also an abnormal increment in the person's BMI will happen inevitably [1, 2].

The World Health Organization (WHO) estimate to Ethiopia denoted that 34% of the annual death rate is attributable to NCD. The greatest proportion (15%) of these deaths is owing to cardiovascular diseases, where DM accounted for the 2% [2]. One small-scale community-based study conducted in Ethiopia to determine the prevalence of known risk factors for NCD reported 9.3% smoking, 7.1% alcohol use, 38.6% khat use, 27.0% poor dietary practice, and 16.9% poor physical exercise. Other than these behavioral risk factors, 9.3% high blood pressure, 2.6% BMI > 25 kg/m2, 10.7% high cholesterol level, and 7.7% raised triglyceride levels were also among the reported physical and biochemical risk factors [4].

Up until now, there is no nationally representative survey conducted to determine the prevalence of hypertension (HTN) and DM in the country [5]. However, there are few small-scale, community- and institution-based studies reporting the magnitude. Seven community-based studies conducted in Ethiopia revealed prevalence of HTN ranging from 9.9% to 28.3% [6–12]. The other reports from three institution-based studies also revealed a similar proportion among adults with HTN prevalence ranging from 7.7% to 27.3% [13–15]. The prevalence of DM in Ethiopia is only emanating from four institution-based survey studies revealing a range of 0.34%-8% [16–19]. These same studies showed that having DM is a risk factor in developing HTN and vice versa. It also demonstrated that they share similar risk factors. These include age, sex, income, physical inactivity, raised BMI, high salt intake, alcohol use, cigarette smoking, and eating vegetable three or fewer days per week.

The Federal Democratic Republic Ministry of Health (FMOH) of Ethiopia affirmed the fact that NCDs are rising at an alarmingly fast rate [5]. Conducting ongoing representative surveys in the area is vital to planning monitoring and evaluation of the effectiveness of health promotion and NCD preventive programs. Nevertheless, there is a paucity of research evidence reporting magnitudes and risk factors of NCD in the country [4, 5]. This study, therefore, is conducted to assess the prevalence of risk factors of NCD, particularly, of HTN and DM, their magnitude and factors associated with these NCDs among patients attending Hawassa University Comprehensive and Specialized Hospital (HU-CSH).

## 2. Methods

This institution-based cross-sectional study was conducted in January 2016 among patients attending Hawassa University Comprehensive Specialized Hospital (HU-CSH). This tertiary level Public University Hospital is the biggest in the region. It serves as a last referral destination to more than 15 million people residing in the region. The hospital provides services partially to people coming from the southern borders of the neighboring Oromia Region of Ethiopia. The HU-CSH is located at the SNNPR's capital, Hawassa City Administration, at 275 km distance south to Addis Ababa. The hospital has six outpatient departments (OPD), namely, Internal Medicine OPD, Surgical OPD, Pediatrics OPD, Obstetrics and Gynecology OPD, Special Clinics and Emergency OPD. We conducted the study mainly on patients receiving service at medical OPD and surgical OPD rooms. During the study period, more than 3,000 people visited the hospital in the specified departments. Of these people, we selected our study participants.

The minimum sample size used in this study was determined using a single population proportion formula. Considering 95 percent confidence interval (CI), alpha level 0.05, a proportion of 0.5%, and 10% nonresponse rate, we determined the minimum sample size of 422 clients. Eleven cases with incomplete data were excluded from the analysis. Therefore, this study is based on 411 patients. Every 8th patient, by using a systematic sampling technique, was included in the study among adult patients attending the selected outpatient department, where K was N/n. In 2014/2016 (2007 Ethiopian Fiscal year), 93,944 OPD patients attended. Patients of at least 18 years old, able to give informed consent and not seriously sick, were included in the study. However, we excluded patients with hearing difficulty, unconscious or with serious mental disability, and pregnant women from the study. Performing of more than 20–30 min of moderate exercises (for instance, hurried walking and/or jogging) for at least four times per week was considered as performing the recommended level of physical activity.

The data were collected by trained nurses using a pretested structured interviewer-administered questionnaire and also observational chart review checklist. The questionnaire was designed to gather the client's sociodemographic data and also variable related risk factors of NCDs such as client's substance use history, questions regarding daily fruit and vegetable consumption status, and client's regular physical exercise history. The observation checklist also contained questions to abstract client's medical diagnosis, treatment, and examination results such as weight and height. The study instrument was prepared by reviewing similar scientific study report [20].

The data collected was first entered, cleaned, and analyzed using the Statistical Package Software for Social Science (SPSS) version 20. By using a univariate descriptive statistical analysis, we determined the frequency and prevalence. The bivariate and multivariate logistic regression analysis model was fitted to determine the Cruds Odds Ratio (COR) and Adjusted Odds Ratio (AOR). All variables with their* P* value < 0.2 were all taken to the next multivariate analysis model. However, those variables demonstrated statistically significant association at* P* value < 0.05 in the multivariate analysis were taken to be factors affecting the dependent variables that are DM and HTN diagnosis.

The quality of the study was assured by pretesting of the questionnaire and also a proper translation of the questionnaire originally prepared in English to Amharic and back to English by two scholars of good language command in both languages. To control the effect of confounders, we used the multivariate logistic regression analysis model.

## 3. Results

### 3.1. Sociodemographic Characteristics of the Study Participants

Nearly, all expected study participants included in the current study successfully responded to the interviewer-administered survey instrument. This makes the nonresponse rate very minimal, which is 2.75%. In this cross-sectional survey, a total of 411 patients, 234 men, and 177 women were studied. Of these, 359 (87.3%) were in the age group of 18–59 years, 268 (65.2%) were urban dwellers, 165 (40.1%) were illiterate, 101 (24.6%) were farmers, and 293(71.3%) were currently living in a marital union (Table 1).

### 3.2. Prevalence of Risk Factors for Hypertension and Diabetes

Concerning the BMI, 83 (20.2%) of the patients had a BMI of at least 25. The majority (98.3) of the patients had been getting vegetables in their daily meals. Alcohol drinking, khat chewing, cigarette smoking, and use of marijuna or Hashish were experienced by 60 (14.6%), 41 (10.0%), 11(2.7%), and 4 (1.0%) patients, respectively. Khat chewing and cigarette smoking were more common among men. However, physical inactivity was more common among women. One hundred fifty-eight (38.4%) of the patients had been experiencing one of these unhealthy lifestyle factors. Patients who experienced 2 and 3 of the unhealthy lifestyle factors constituted 71 (17.3%) and 21 (5.1%), respectively (Table 2).

**Figure 1:**
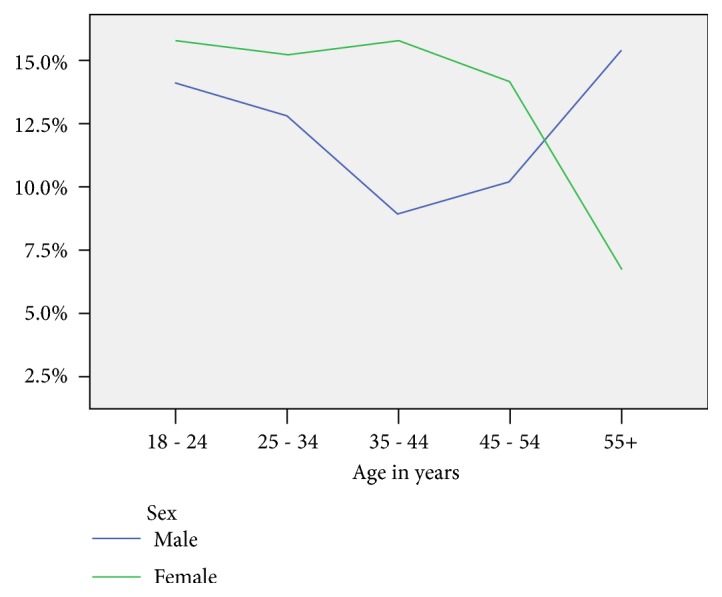
Pattern of any of the risk factors of Hypertension and Diabetes by age group and sex among patients at HU-CSH, January 2015.

The pattern of the risk factors among women reduced as age increased. Among men, as age increased up to 35–40 years, the magnitude of risk factors reduced; then the risk factors raised with age, from around 14% among age group 35–45 to more than 24% among 55+ years age group.  Figure 1 shows the pattern of risk factors of hypertension and diabetes mellitus by age group and sex (Figure 1).

Among the study participants, the prevalence of hypertension, prevalence of diabetes mellitus, and prevalence of both hypertension and diabetes mellitus were 10.5%, 12.2%, and 3.2, respectively. The prevalence of hypertension and or diabetes mellitus was 19.5% (Table 2). Prevalence of hypertension and/or DM steadily increased from below 10% among 25–34 years age group to nearly 35% among patients in the age group of 55+ years.

In unadjusted regression analysis age, smoking, khat chewing, alcohol drinking, and BMI were associated with having of hypertension. In the multivariate analysis, old age (AOR = 4.0, 95% CI 1.8–9.0), smoking (AOR = 5.0, 95% CI 1.1–23.3), and high BMI (AOR = 5.7, 95% CI 2.7–11.9) maintained the significance in predicting having of hypertension (Table 3).

Concerning diabetes mellitus, exercise, alcohol drinking, khat chewing, and BMI were associated with having diabetes mellitus in unadjusted analysis. In a multivariate logistic regression analysis, only exercise (Adj. OR = 3.0, 95% CI 1.6–5.7) and high BMI (Adj. OR = 4.9, 95% CI 2.5–9.7) were found to predict having diabetes mellitus. Details on factors determining diabetes mellitus are described in Table 4.

## 4. Discussion

In this study, we found high prevalence DM and HTN. Unhealthy life style was experienced by significant proportion of the study participants. Variables like age, smoking, and BMI were associated with having of hypertension, while exercise and BMI predicted having DM.

The finding of this study revealed a 12.2% prevalence of DM among clients attending the OPD/s of HU-CSH. Four institution-based cross-sectional study reports from Ethiopia reported a prevalence of DM ranging from 0.32% to 8.0%. These are 5.0% among Addis Ababa Police officers [16], 0.34% among patients attending Debre Berhan Hospital [17], 8.0% among Gondar University Hospital [18], and 6.4% among Jimma University Hospital [19]. The latter two studies were conducted on similar university hospitals which are comparable to the current study site. However, they were conducted merely on HIV-infected clients. In contrast to these study reports and also the global prevalence of DM (8.8%), the prevalence obtained in the current study is higher. The differences noted in these reports might be linked to the type of clients involved in these studies and the peculiar geographic and sociodemographic characteristics. In addition, the methods and settings used in the study were all reasons to justify the observed differences.

Hypertension is the most common reason of physician visit among adults in USA. The estimated prevalence of HTN among US citizens ranges from 29 to 30% [21]. Nearly, in a similar fashion, the existing few institution- and community-based studies from Ethiopia reported comparable prevalence of hypertension. As a literature review from two studies denoted, the prevalence of hypertension in Ethiopia ranges from 7.7% to 28.3% [11, 14]. This is in agreement with the WHO report that the fast increment of the magnitudes of NCDs is in the developing countries [1, 2].

In the current study, however, we identified relatively lower prevalence of HTN. This finding is similar to one community-based study conducted in a nearby zone called Sidama Zone, which reported a 9.9% HTN and another institution-based study reported a 13.2% prevalence of HTN among clients attending at Jimma University Referral Hospital [12, 15]. The only small, but logically acceptable figure reported from Ethiopia, HTN prevalence of 7.7%, is from undergraduate Gondar University students. The current study finding, nevertheless, is by far lesser from a report of a study conducted among federal ministry civil servants in Addis Ababa [13].

In this study, we identified prevalence for four substances: alcohol 14.6%, khat chewing 10.7%, cigarette 2.7%, and hashish 1.0%. The prevalence of alcohol in this study is high, but seems to be lower compared to other study reports which reported an average of nearly 50% [22, 23]. Khat is locally cultivated evergreen shrub containing psychoactive ingredients called cathine and cathinone. It is one of the known risk factors of cardiovascular diseases especially for HTN and ischemic heart diseases. [24, 25]. In another study, the prevalence of khat chewing varied from 13.4% to 41.0% [26]. The existing prevalence of cigarette smoking in Ethiopia is 7.7%. In contrast to the current study finding, these reports seem lower [27, 28]. However, the prevalence identified in the current study still is in a level of significant public health problem.

The prevalence of other risk factors of NCD identified in our study included a 20.2% BMI of ≥ 25, 33.3% physical inactivity, and substandard consumption of fruit and or vegetables of 1.7%. The latter is by far negligible compared to a study conducted in Ethiopia which reported 27.0% and the WHO multinational survey reported 72% prevalence of substandard fruit and vegetable consumption [4, 29]. This finding can be explained by the local production and availability of fruits and vegetables in abundance. However, the proportion of people who are overweight and/or obese with their BMI score ≥ 25 reported in the current study is nearly comparable to the report from Addis Ababa [13], but higher than that in other reports [4, 30].

The level of physical inactivity found in this study is higher than that in other reports [4, 30, 31]. The rapid urbanization, the increasing tendency of using a motor vehicle for daily transportation and communities' abandonment of bicycle use trend, might have contributed to the identified physical inactivity. The higher physical inactivity noted in the current study may also explain the observed higher BMI reported in this same population [32]. As it is shown in Figure 1, the prevalence of any of the risk factors is more common in women of the younger age group. However, it is higher among male in the elderly age group. The higher prevalence of any of the risk factors among women in the younger age group could be due to the fact that physical inactivity and a BMI score of > 25 are more common among women than men.

As age increases, the likelihood of developing HTN also increases. Unfortunately, older age is one of the unavoidable risk factors to developing HTN [9, 12]. The current study also identified older age as one of the risk factors. The likelihood of developing HTN was by fourfold higher than among those aged > 60 years (AOR= 4.0, 95% CI 1.8 -9.0). This finding may serve as an evidence to support interventions promoting the need for regular HTN screening checkup [5, 13]. However, cigarette smoking and higher BMI were among the proven avoidable or modifiable risk factors identified in the current study for increasing the risk of developing HTN by five- and sixfold, respectively. These findings are consistent with other study results reported from Ethiopia and the world [9, 12, 13].

The current study result showed that higher BMI was a factor increasing the likelihood of developing DM. The risk of developing DM as a result of a higher BMI among the study population was five times higher than those with lower BMI. This finding is in agreement with other study reports showing higher BMI as the most important predictor of DM [17, 32]. The chance of developing DM increases by threefold among physically inactive patients. The risk of physical inactivity for developing DM is a well-known risk factor which supports the current study finding [33]. Performing regular exercise along with proper nutrition is the best means to control the BMI within the normal limit and prevent DM [32, 33].

## 5. Limitation of the Study

This study is the first for the area which may serve to lay a baseline finding pertaining to prevalence of risk factors and associated factors with HTN and DM. However, its cross-sectional nature makes it insufficient to establish a temporal relationship between the factors and the diseases conditions. Participants of the study might not remember to respond accurately to some of the questions. Since we conducted an institution-based study, generalization of the study findings to other setups need a meticulous consideration.

## 6. Conclusion

The prevalence of DM among clients attending at HU-CSH was high. The prevalence of HTN was also substantial. Higher BMI, physical inactivity, low fruit and vegetable consumption, alcohol use, khat chewing, and cigarette smoking were among the prevailing risk factors identified. Older age, higher BMI, and physical inactivity were among the factors increasing the risks of developing HTN. Having a higher BMI and physical inactivity were factors found increasing the risk of developing DM among the studied population. The magnitudes of DM and HTN are at a considerable level indicating the need for further strengthening the existing preventive works. This study also demonstrated avoidable or modifiable risk factors leading to HTN and DM.

## Figures and Tables

**Table 1:** Sociodemographic characteristics of the study participants attending university teaching hospital at Hawassa, Southern Ethiopia, 2016.

Sociodemographic characteristics	Male		Female		Total	
	Freq. (n)	Per. (%)	Freq. (n)	Per. (%)	Freq. (n)	Per. (%)
*Age*						
18-59	192	82.1	167	94.4	359	87.3
≥ 60	42	17.9	10	5.6	52	12.7
*Residence*						
Urban	138	59.0	130	73.4	268	65.2
Rural	96	41.0	47	26.6	143	34.8
*Occupation*						
Employed	59	25.2	42	23.7	101	24.6
Farmer	89	38.0	10	5.6	99	24.1
Housewife	0	0	74	41.8	74	18.0
Student	43	18.4	27	15.3	70	17.0
Other	43	18.4	24	13.6	67	16.3
*Marital status*						
Currently in marital union	163	69.7	130	73.4	293	71.3
Currently not in marital union	71	30.3	47	26.6	118	28.7
*Education*						
Literate	146	62.4	100	56.5	246	59.9
Illiterate	88	37.6	77	43.5	165	40.1
*Household income *						
≥ 1500	135	57.7	94	53.1	229	55.7
< 1500	99	42.3	83	46.9	182	44.3

**Table 2:** Prevalence of risk factors of HTN and DM, types of chronic diseases, and number of risk factors by patients at HU-CSH, January 2015.

Factors	Male; n (%)	Female; n (%)	Total; n (%)
*Types of identified risk factors*			

Clients with any of the risk factors	144 (61.5)	120 (67.8)	264 (64.2)
Physical inactivity*∗*	58 (24.8)	79 (44.6)	137 (33.3)
BMI high ≥ 25	40 (17.1)	43(24.3)	83 (20.2)
Alcohol drinking	52(22.2)	8(4.5)	60 (14.6)
Khat chewing*∗*	34 (14.5)	7 (4.0)	41 (10.0)
Smoking cigarette*∗*	11 (4.7)	0 (0.0)	11 (2.7)
Substandard consumption of fruit and or vegetables	3 (1.3)	4 (2.3)	7 (1.7)
Hashish	4 (1.7)	0 (0.0)	4 (1.0)

*Type of chronic diseases*			

Hypertension and or diabetes mellitus (n = 411)	46 (19.7)	34 (19.2)	80 (19.5)
Diabetes mellitus (n = 411)	26 (11.1)	24 (13.6)	50 (12.2)
Hypertension (n = 411)	26 (11.1)	17 (9.6)	43 (10.5)
Hypertension and diabetes mellitus (n = 411)	6 (2.6)	7 (4.0)	13 (3.2)

Unhealthy lifestyle = BMI >/= 25, BMI = <18, not exercising, not taking fruit/vegetables, alcohol drinking, khat chewing, cigarette smoking, hashish taking; *∗*variables with statistically significant difference by gender.

**Table 3:** Risk factors of hypertension among patients at HU-CSH, January 2015.

Variables		Yes	No	UOR (95% CI)	AOR (95% CI)
Age in years	*18-59*	30	329		
	*≥ 60*	13	39	3.7 (1.8 – 7.6)*∗*	4.0 (1.8 – 9.0)*∗*

Household income	*≥ 1500*	30	199		
	*< 1500*	13	169	0.5 (0.3 – 1.0)	0.9 (0.4 – 1.9)

Smoking	*No*	39	361		
	*Yes*	4	7	3.6 (1.1 – 12.1)*∗*	5.0 (1.1 – 23.3)*∗*

Khat chewing	*No*	34	336		
	*Yes*	9	32	2.8 (1.2 – 6.3)*∗*	1.3 (0.4 – 4.2)

Alcohol drinking	*No*	30	321		
	*Yes*	13	47	3.0 (1.4 – 6.1)*∗*	1.5 (0.6 – 3.9)

BMI	*14 – 25*	20	308		
	*High*	23	60	5.9 (3.1 – 11.4)*∗*	5.7 (2.7 – 11.9)*∗*

*∗P* < 0.05, UOR: unadjusted odds ratio, AOR: adjusted odds ratio, CI: confidence interval.

**Table 4:** Risk factors of diabetes mellitus among patients at HU-CSH, January 2015.

Variables		Yes	No	UOR (95% CI)	AOR (95% CI)
Residence	*Urban*	37	231		
	*Rural*	13	130	0.6 (0.3 – 1.2)	0.8 (0.4 – 1.7)

Household income	*≥ 1500*	33	196		
	*<1500*	17	165	0.6 (0.3 – 1.1)	0.8 (0.4 – 1.7)

Exercise	*Yes*	24	250		
	*No*	26	111	2.4 (1.3 – 4.4)*∗*	3.0 (1.6 – 5.7)*∗*

Alcohol drinking	*No*	37	314		
	*Yes*	13	47	2.3 (1.2 – 4.7)*∗*	1.4 (0.6 – 3.5)

Khat chewing	*No*	40	330		
	*Yes*	10	31	2.7 (1.2 – 5.8)*∗*	1.7 (0.6 – 4.6)

BMI	*14 – 25*	25	303		
	*Low or high*	25	58	5.2 (2.8 – 9.7)*∗*	4.9 (2.5 – 9.7)*∗*

*∗P* < 0.05, UOR: unadjusted odds ratio, AOR: Adjusted odds ratio, CI: confidence interval.

## Data Availability

The data used to support the findings of this study are included within the article.

## Additional Points

Noncommunicable diseases are the cause of 63% of annual global death report, of which 14 million deaths are premature. Nearly, all (86%) of the premature deaths are happening in the middle- and lower-income countries (LMICs), including Ethiopia. Eighty percent (80%) of the CVD and DM can be prevented by avoiding the behavioral risk factors, such as tobacco use, physical inactivity, unhealthy dietary practice, and alcohol abuse. This study, as its contribution, determined the prevalence of DM and HTN among clients attending to the outpatient departments of the biggest hospital in Southern Ethiopia. In this study, the prevalence of the risk factors and the risk factors association with the development of DM and HTN were all identified. These findings can inform policy makers, clinicians, and researchers to work towards curbing the existing situation by considering the current findings.
